# Screening potential lncRNA biomarkers for breast cancer and colorectal cancer combining random walk and logistic matrix factorization

**DOI:** 10.3389/fgene.2022.1023615

**Published:** 2023-01-20

**Authors:** Shijun Li, Miaomiao Chang, Ling Tong, Yuehua Wang, Meng Wang, Fang Wang

**Affiliations:** Department of Pathology, Chifeng Municipal Hospital, Chifeng, China

**Keywords:** breast cancer, colorectal cancer, lncRNA, biomarker, lncRNA-disease association, random walk, logistic matrix factorization

## Abstract

Breast cancer and colorectal cancer are two of the most common malignant tumors worldwide. They cause the leading causes of cancer mortality. Many researches have demonstrated that long noncoding RNAs (lncRNAs) have close linkages with the occurrence and development of the two cancers. Therefore, it is essential to design an effective way to identify potential lncRNA biomarkers for them. In this study, we developed a computational method (LDA-RWLMF) by integrating random walk with restart and Logistic Matrix Factorization to investigate the roles of lncRNA biomarkers in the prognosis and diagnosis of the two cancers. We first fuse disease semantic and Gaussian association profile similarities and lncRNA functional and Gaussian association profile similarities. Second, we design a negative selection algorithm to extract negative LncRNA-Disease Associations (LDA) based on random walk. Third, we develop a logistic matrix factorization model to predict possible LDAs. We compare our proposed LDA-RWLMF method with four classical LDA prediction methods, that is, LNCSIM1, LNCSIM2, ILNCSIM, and IDSSIM. The results from 5-fold cross validation on the MNDR dataset show that LDA-RWLMF computes the best AUC value of 0.9312, outperforming the above four LDA prediction methods. Finally, we rank all lncRNA biomarkers for the two cancers after determining the performance of LDA-RWLMF, respectively. We find that 48 and 50 lncRNAs have the highest association scores with breast cancer and colorectal cancer among all lncRNAs known to associate with them on the MNDR dataset, respectively. We predict that lncRNAs HULC and HAR1A could be separately potential biomarkers for breast cancer and colorectal cancer and need to biomedical experimental validation.

## 1 Introduction

Breast cancer is the second leading cause of cancer-related death in women worldwide and the most common malignant tumor among US woman (Sun et al., 2017; DeSantis et al., 2019; Yang et al., 2013; Waks and Winer, 2019). During the past 25 years, breast cancer mortality rate showed a substantial increase in the world (Garrido-Castro et al., 2019). This increasing rate is one threaten to health for women in the world, in particular women from developing and low-income regions. More than 1.5 million women were diagnosed to breast cancer every year, which accounts for 25% among all women with cancers (Sun et al., 2017). In 2018, breast cancer accounts for approximately 24% of new cancer cases and approximately 15% of cancer deaths in women (Heer et al., 2020). In 2019, it is estimated that about 268,600 new patients suffer from invasive breast cancer and 48,100 patients suffer from ductal carcinoma *in situ* among US women. Moreover, 41,760 women may die from breast cancer in the same year (DeSantis et al., 2019). About 13% of women may suffer from invasive breast cancer in lifetime (DeSantis et al., 2019). The incident rate of breast cancer will increase by more than 46% by 2040 (Heer et al., 2020). Consequently, breast cancer has been one essential problem to be solved around the world.

However, the precise mechanisms of breast cancer remain unclear (Barzaman et al., 2020). Systemic treatment of breast cancer patients mainly consists of chemotherapy, endocrine treatment, and targeted therapy (Campos-Parra et al., 2018). In spite of rapid progress in different treatment strategies, accumulating patients show recurrence of the disease and decreased survival because of therapy resistance, which increases metastasis rates (Sledge et al., 2014). Once the metastasis occurs, the 5-year overall survival rate may be below 25% (Siegel et al., 2013).

Colorectal cancer is the third most frequent cancer and the second most death-caused cancer. It is estimated that there are about 1.9 million new cases and 0.9 million death cases worldwide in 2020 (Xi and Xu, 2021). Of new diagnose cases, 20% of patients have metastases and another 25% with localized disease may later develop metastases (Biller and Schrag, 2021). Its incidence is high in developed countries and is increasing in low- and middle-income countries, which poses a challenge to global public health (Biller and Schrag, 2021; Xi and Xu, 2021).

In this situation, it is essential to discover novel molecular biomarkers that can characterize therapy response for breast cancer and colorectal cancer. We can extend the overall survival rates of patients and delay or prevent the two cancers from metastases based on molecular biomarkers (Campos-Parra et al., 2018). Consequently, screening reliable biomarker is a research hotspot on the diagnosis and treatment of cancer including breast cancer and colorectal cancer (Huang et al., 2019; Yang et al., 2020; Peng et al., 2022a).

A substantial number of evidence suggest that over 80% of the human genome can be transcribed into non-coding RNAs, such as microRNAs (Peng et al., 2017; Peng et al., 2018; Chen et al., 2019; Huang et al., 2021), circle RNAs (Zhao et al., 2019; Lan et al., 2022), and long non-coding RNAs (lncRNAs) (Zhang et al., 2021a; Peng et al., 2021a; Peng et al., 2022b; Zhou et al., 2021a; Zhou et al., 2021b). In particular, lncRNAs obtain emerging interest as diagnostic biomarkers and therapeutic targets (Chandra Gupta and Nandan Tripathi, 2017; Guo et al., 2022). Differential expression of lncRNAs forms specific patterns to various complex diseases including cancer (Wahlestedt C, 2013). Once the regulation effects of lncRNAs are detected, they are promising therapeutic targets.

LncRNAs are closely related to breast cancer and colorectal cancer. For example, lncRNA BCRT1, MaTAR25, DSCAM-AS1, and CDC6 can promote breast cancer progression (Niknafs et al., 2016; Kong et al., 2019a; Chang et al., 2020; Liang et al., 2020), BCRT4 can induce signaling transduction in breast cancer (Xing et al., 2015), LINC00673 can promote cell proliferation of breast cancer (Qiao et al., 2019), and BORG can cause breast cancer metastasis and disease recurrence (Gooding et al., 2017). SNHG11, FEZF1-AS1, RP11, and DLEU1 have been reported to novel biomarkers of colorectal cancer (Bian et al., 2018; Liu et al., 2018; Wu et al., 2019; Xu et al., 2020). Thus, many computational models have been developed to discover lncRNA biomarkers for cancers (Peng et al., 2020a; Shen et al., 2022; Sun et al., 2022), for instance, rotation forest (Guo et al., 2019), KATZ measure (Chen, 2015), collaborative deep learning (Lan et al., 2020), matrix factorization (Fu et al., 2018; Wang et al., 2021a), network consistency projection (Li et al., 2019), and graph autoencoder (Shi et al., 2021).

In this manuscript, inspired by the association prediction method provided by Peng et al. (2020b), we develop a computational method, LDA-RWLMF, to predict LncRNA-Disease Associations (LDAs). LDA-RWLMF integrates random walk and Logistic Matrix Factorization to discover the roles of lncRNA biomarkers in the prognosis and diagnosis for breast cancer and colorectal cancer. First, we compute disease similarity and lncRNA similarity. Second, we first use random walk to extract negative LDAs. Third, we explored a logistic matrix factorization model to predict possible LDAs. The results from 5-fold cross validation show that LDA-RWLMF computes the best AUC value of 0.9312 on the MNDR dataset. Finally, we rank all lncRNA biomarkers for breast cancer and colorectal cancer after determining the performance of LDA-RWLMF.

## 2 Datasets

### 2.1 LncRNA-disease associations

Human LDA dataset was collected from the MNDR database (Cui et al., 2018; Fan et al., 2020) (http://www.rna-society.org/mndr/index.html). There are 1,529 LDAs between 89 diseases and 190 lncRNAs after preprocessing. For an LDA matrix between n lncRNAs and m diseases, we use $Y\epsilon R^{n\times m}$ to describe the association information by Eq. 1:

$$Y_{ij}=\left\{ \begin{array}{ll} 1\;If\;lncRNA\;l_{i} & associates\;with\;d_{j}\\ 0 & otherwise \end{array}\right.$$ (1)

### 2.2 Disease semantic similarity

We use the method provided by Fan et al. (2020) to compute disease semantic similarity based on the MeSH descriptors. Disease semantic similarity method provided by Fan et al. (2020) was based on LNCSIM1 and LNCSIM2 provided by Chen (2015). For a disease A , suppose that $T_{A}$ represents its ancestor node set, $E_{A}$ denotes all edge set, its Directed Acyclic Graph (DAG) is represented as ${DAG}_{A}=\left\{T_{A},E_{A}\right\}$ . For a disease term $t\in T_{A}$ in ${DAG}_{A}$ , its semantic contribution to A is calculated by Eq. 2 (Chen, 2015):

$${SV}_{A}^{1}\left(t\right)=\left\{ \begin{array}{ll} 1 & t=A\\ \max(∆\times {SV}_{A}^{1}\left(t^{\prime }\right)|t^{\prime }\in C\left(t\right) & t\neq A \end{array}\right.$$ (2)

where C(t) indicates the children of t , ∆ indicates the sematic contribution factor related to edges that link $t^{\prime }$ to t , and ∆ was usually set as 0.5 (Wang et al., 2010).

The above equation demonstrates that terms at the same layer from ${DAG}_{A}$ have the same semantic contribution to A . But if two terms $t_{1}$ and $t_{2}$ are in the same layer of ${DAG}_{A}$ and $t_{1}$ appears in less in ${DAG}_{A}$ than $t_{2}$ , the conclusion from $t_{1}$ will be more specific than one from $t_{2}$ , thus, ${SV}_{A}^{1}\left(t_{1}\right)$ is higher than ${SV}_{A}^{1}\left(t_{2}\right).$

In this case, we compute the second semantic contribution of term $t\in T_{A}$ to disease A by Eq. 3:

$${SV}_{A}^{2}\left(t\right)=-\log\frac{Dags\left(t\right)}{D}$$ (3)

where D indicates the number of diseases in MeSH, Dags(t) indicates the number of DAGs that contain the disease term t . And the semantic contribution of t in ${DAG}_{A}$ can be defined by Eq. 4:

$${SV}_{A}^{3}\left(t\right)=\left\{ \begin{array}{cc} 1 & t=A\\ \max((∆+\nabla ){SV}_{A}^{3}\left(t^{\prime }\right)|t^{\prime }\in C\left(t\right) & t\neq A \end{array}\right.$$ (4)

where ∇ indicates the contribution factor related to information content, and is computed by Eq. 5:

$$\nabla =\frac{\max_{k\in K}\left(Dags\left(k\right)\right)-dags\left(t\right)}{D}$$ (5)

where K indicates the disease set in MeSH.

Furthermore, the contribution of all terms in ${DAG}_{A}$ to the disease A is computed by Eq. 6:

$$SV\left(A\right)=\underset{t\in T_{A}}{\sum }{SV}_{A}^{3}\left(t\right)$$ (6)

Finally, the semantic similarity between two diseases (*A* and *B*) can be computed by Eq. 7:

$$S_{d}^{s}\left(A,B\right)=\frac{\sum_{t\in T_{A}\cap T_{B}}\left(SV_{A}^{3}\left(t\right)+SV_{B}^{3}\left(t\right)\right)}{SV\left(A\right)+SV\left(B\right)}$$ (7)

### 2.3 LncRNA functional similarity

We use the method provided by Fan et al. (Fan et al., 2020) to compute lncRNA functional similarity. Let that *DG*(*u*) [or *DG*(*v*)] indicate diseases linking to lncRNA u (or v ) on LDA matrix, the similarity between two lncRNAs u and v is obtained through disease semantic similarity in *DG* (*u*) and *DG* (*v*). A disease semantic similarity sub-matrix is first constructed. In the constructed matrix, rows and columns are diseases in *DG* (*u*) ∪*DG* (*v*), and each element indicates the semantic similarity between diseases. Suppose that $d_{u}$ indicate a disease in *DG* (*u*), the similarity between $d_{u}$ and *DG*(*v*) is computed by Eq. 8:

$$S\left(d_{u},DG\left(v\right)\right)=\max_{d\in DG\left(v\right)}\left(S_{d}\left(d_{u},d\right)\right)$$ (8)

Similarly, the similarity between $d_{v}$ and *DG* (*u*) is computed by Eq. 9:

$$S\left(d_{v},DG\left(u\right)\right)=\max_{d\in DG\left(u\right)}\left(S_{d}\left(d_{v},d\right)\right)$$ (9)

And the similarity of DG(u)→DG(v) is computed by Eq. 10:

$$S_{u\to v}=\underset{d\in DG\left(u\right)}{\sum }S\left(d,DG\left(v\right)\right)$$ (10)

And similarity of DG(v)→DG(u) is computed by Eq. 11:

$$S_{v\to u}=\underset{d\in DG\left(v\right)}{\sum }S\left(d,DG\left(u\right)\right)$$ (11)

The similarity between lncRNAs u and v is measured based on the disease semantic similarity by Eq. 12:

$$S_{l}^{f}\left(u,v\right)=\frac{S_{u\to v}+S_{v\to u}}{\left|DG\left(u\right)\right|+\left|DG\left(v\right)\right|}$$ (12)

where |DG(u)| and |DG(v)| are the number of diseases in DG(u) and DG(v) .

## 3 Methods

**FIGURE 1:**
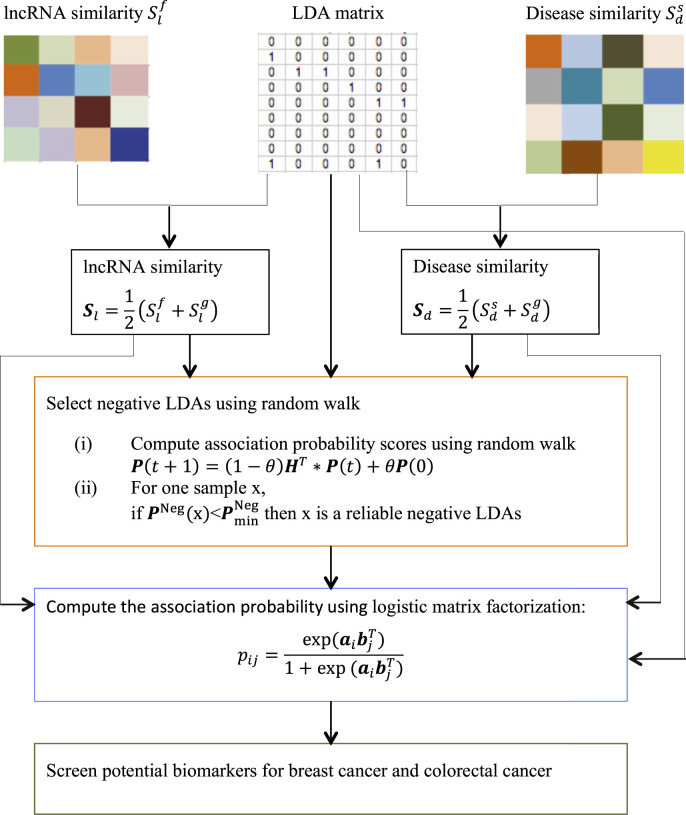
The pipeline of LDA-RWLMF.

We want to compute association probability for each lncRNA-disease pair based on disease semantic similarity and lncRNA functional similarity. The pipeline is shown in Figure 1.

### 3.1 Gaussian association profile similarity and similarity fusion

In this section, we use Gaussian Association Profile (GAP) to compute the GAP similarity of diseases and lncRNAs. For a lncRNA $l_{i}$ , its GAP $AP\left(l_{i}\right)$ is denoted using the i th row of ***Y***. The GAP similarity of lncRNAs $l_{i}$ and $l_{j}$ is defined by Eq. 13:

$$S_{l}^{g}\left(l_{i},l_{j}\right)=\exp\left(-\gamma_{l}{\left\|AP\left(l_{i}\right)-AP\left(l_{j}\right)\right\|}^{2}\right)$$ (13)

where $\gamma_{l}=\gamma_{l}^{\prime }/\left(\frac{1}{n}\;\overset{n}{\underset{k=1}{\sum }}\;∥AP\left(l_{i}\right){∥}^{2}\right)$ is the normalized kernel bandwidth with parameter $\gamma_{l}^{\prime }$ . Thus, the lncRNA similarity matrix $S_{l}$ is computed by Eq. 14:

$$S_{l}=\frac{1}{2}\left(S_{l}^{f}+S_{l}^{g}\right)$$ (14)

Similarly, the disease GAP similarity $S_{d}$ can be computed.

### 3.2 Screening negative LDAs

There are not negative LDAs in the MNDR dataset. Credible negative LDAs help improve LDA prediction performance and further more effectively find potential lncRNA biomarkers for breast cancer and colorectal cancer. Peng et al. (2021b) developed a random walk with restart-based virus-drug association prediction method and obtained better performance. Inspired by the method provided by Peng et al. (2021b), we first compute association probability for each lncRNA-disease pair through random walk with restart and then screen credible negative LDAs.

We first constructed a heterogeneous network composed of lncRNA similarity network, disease similarity network, and LDA network. lncRNA similarity matrix $S_{l}$ , disease similarity matrix $S_{d}$ , and LDA matrix Y are used as the adjacency matrices related to the heterogeneous network. The adjacency matrix related to the heterogeneous network is represented as Eq. 15:

$$H=\left[\begin{array}{cc} S_{l} & Y\\ Y^{T} & S_{d} \end{array}\right]$$ (15)

where $Y^{T}$ denotes the transpose of Y .

We then compute transition probability on the heterogeneous graph. Suppose that $H=\left[\begin{array}{cc} H_{ll} & H_{ld}\\ H_{dl} & H_{dd} \end{array}\right]$ indicate transition probability matrix, where $H_{ll}$ and $H_{dd}$ indicate the walks within lncRNA similarity network and disease similarity network, respectively, $H_{ld}$ and $H_{dl}$ indicate the jumps between networks. For an lncRNA/disease, when there is an association between the lncRNA/disease and diseases/lncRNAs, the node will either continue to walk in the current network based on a transition probability λ∈[0,1] or jump between the above four networks.

The i -th lncRNA will walk to the j -th lncRNA through the transition probability $H_{ll}\left(i,j\right)$ by Eq. 16:

$$H_{ll}\left(i,j\right)=\left\{ \begin{array}{cc} \frac{S_{l}\left(i,j\right)}{\overset{n}{\underset{k=1}{\sum }}\;S_{l}\left(i,k\right)}, & if\;\sum_{k=1}^{m}\;Y\left(i,k\right)=0\\ \frac{\left(1-\lambda \right)S_{l}\left(i,j\right)}{\overset{n}{\underset{k=1}{\sum }}\;S_{l}\left(i,k\right)}, & otherwise \end{array}\right.$$ (16)

or jump to a disease $d_{j}$ through the transition probability $H_{ld}\left(i,j\right)$ by Eq. 17:

$$H_{ld}\left(i,j\right)=\left\{ \begin{array}{l} \frac{\lambda Y\left(i,j\right)}{\overset{m}{\underset{k=1}{\sum }}\;Y\left(i,k\right)},\;if\;\sum_{k=1}^{m}\;Y\left(i,k\right)\neq 0\\ 0,\;otherwise \end{array}\right.$$ (17)

Similarly, the i -th disease $d_{i}$ will walk to the j -th disease $d_{j}$ through the transition probability $H_{dd}\left(i,j\right)$ by Eq. 18:

$$H_{dd}\left(i,j\right)=\left\{ \begin{array}{ll} \frac{S_{d}\left(i,j\right)}{\overset{m}{\underset{k=1}{\sum }}\;S_{d}\left(i,k\right)}, & if\;\overset{n}{\underset{k=1}{\sum }}\;Y\left(k,i\right)=0\\ \frac{\left(1-\lambda \right)S_{d}\left(i,j\right)}{\overset{m}{\underset{k=1}{\sum }}\;S_{d}\left(i,k\right)}, & otherwise \end{array}\right.$$ (18)

or jump to an lncRNA $l_{j}$ through the transition probability $H_{dl}\left(i,j\right)$ by Eq. 19:

$$H_{dl}\left(i,j\right)=\left\{ \begin{array}{l} \frac{\lambda Y\left(i,j\right)}{\overset{n}{\underset{k=1}{\sum }}\;Y\left(k,i\right)},\;if\;\sum_{k=1}^{m}\;Y\left(k,i\right)\neq 0\\ 0,\;\;\;\;\;otherwise \end{array}\right.$$ (19)

At the t− th step, the association probability matrix between all lncRNA-disease pairs on the heterogeneous network is computed by Eq. 20:

$$P\left(t+1\right)=\left(1-\theta \right)H^{T}*P\left(t\right)+\theta P\left(0\right)$$ (20)

where $H^{T}$ indicates the transpose of H , and θ is the restarting probability. P(0) indicates the initial probability with $p_{i}\left(0\right)=\left[\begin{array}{c} \left(1-\eta \right)v_{i}\\ \eta s_{i} \end{array}\right]$ , where $v_{i}$ and $s_{j}$ indicate the initial probability distributions on disease similarity network and lncRNA similarity network, respectively. And η∈[0,1] is used to control the restarting probability in these two similarity networks. If η<0.5 , the particle will more tend to restart from one of the seed microbes than from one of the seed diseases.

In the second step, we consider known LDAs as positive sample set P , unknown lncRNA-disease pairs as unlabeled set U and propose a PU learning approach to screen credible negative LDA sample set RN . The method contains the following six steps:

Step 1. Randomly screening positive sample subset D from P

Step 2. Adding D into U ;

Step 3. Considering P−D as positive samples, U+D as negative samples;

Step 4. Obtaining LDA score matrix $S^{Neg}$ using random walk with restart;

Step 5. Ranking lncRNA-disease pairs in D based on $S_{\min}^{Neg}$ and obtaining the minimum score $S_{\min}^{Neg}$ in D ;

Step 6. For every lncRNA-disease pair x in U :

If $S^{Neg}\left(x\right)<S_{\min}^{Neg}$ then RN=RN∪x .

### 3.3 LDA prediction based on logistic matrix factorization

Logistic matrix factorization has been applied to multiple areas (Liu et al., 2020; Tang et al., 2021; Tian et al., 2022). Inspired by the approaches, we develop a logistic matrix factorization-based LDA prediction method, LDA-RWLMF.

Assume that both lncRNAs and diseases are mapped to r -dimensional shared latent spaces ( r≪n,m ), thus an lncRNA $l_{i}$ or disease $d_{i}$ can be represented as a latent vector $a_{i}\in R^{1\times t}\;or\;b_{i}\in R^{1\times t}.$ The association probability $p_{ij}$ between $l_{i}$ and $d_{i}$ is calculated by Eq. 12:

$$p_{ij}=\frac{\exp\left(a_{i}b_{j}^{T}\right)}{1+\exp\left(a_{i}b_{j}^{T}\right)}$$ (21)

The latent vector matrix of all lncRNAs or diseases can be represented as $A\in R^{n\times r}or\;B\in R^{m\times r}$ where $a_{i}\;or\;b_{i}$ indicates the i th or j th row in A or B . In addition, known LDAs are more credible than unknown lncRNA-disease pairs. Thus, we assign higher confidence values to known LDAs than unknown lncRNA-disease pairs. Similar to Peng et al. (2020b), we use a constant c to assess the importance of known LDAs and construct a prediction model by Eq. 22:

$$p\left(Y|A,B\right)=\begin{array}{c} \left(\prod_{1\leq i\leq n,1\leq j\leq m,y_{ij}=1}\;{\left[p_{ij}^{y_{ij}}{\left(1-p_{ij}\right)}^{\left(1-y_{ij}\right)}\right]}^{c}\right)\\ \times \left(\prod_{1\leq i\leq n,1\leq j\leq m,y_{ij}=0}\left[p_{ij}^{y_{ij}}{\left(1-p_{ij}\right)}^{\left(1-y_{ij}\right)}\right]\right)\\ =\overset{n}{\underset{i=1}{\prod }}\;\overset{m}{\underset{j=1}{\prod }}\;p_{ij}^{cy_{ij}}{\left(1-p_{ij}\right)}^{\left(1-y_{ij}\right)} \end{array}$$ (22)

Model (21) can be optimized based on the Bayesian distribution by Eq. 23:

$$\min_{A,B}\overset{m}{\underset{i=1}{\sum }}\;\overset{n}{\underset{j=1}{\sum }}\left(1+cy_{ij}-y_{ij}\right)\log\left[1+\exp\left(a_{i}b_{j}^{T}\right)\right]-cy_{ij}a_{i}b_{j}^{T}+\frac{\lambda_{l}}{2}∥A{∥}_{F}^{2}+\frac{\lambda_{d}}{2}∥B{∥}_{F}^{2}$$ (23)

where $\lambda_{l}$ and $\lambda_{d}$ are two parameters, ${\left\|A\right\|}_{F}$ indicates the Frobenius norm of A . (Zhang et al. 2019a; Zhang et al. 2019b) integrated linear neighborhood information to model (22) to predict various associations. Similarly, we fuse neighborhood information to Eq. 23 by Eq. 24:

$$\begin{array}{c} \min_{A,B}\overset{m}{\underset{i=1}{\sum }}\;\overset{n}{\underset{j=1}{\sum }}\left(1+cy_{ij}-y_{ij}\right)\ln\left[1+\exp\left(a_{i}b_{j}^{T}\right)\right]-cy_{ij}a_{i}b_{j}^{T}\\ +\frac{1}{2}tr[A^{T}\left(\lambda_{l}I+\alpha L_{l}\right)A+\frac{1}{2}tr[B^{T}\left(\lambda_{d}I+\alpha L_{d}\right)B \end{array}$$ (24)

where tr (·) indicates the trace of the matrix. $L_{l}$ and $L_{d}$ indicate the corresponding Laplacian matrix of A and B . $L_{l}=\left(D_{l}+{\tilde{D}}_{l}\right)-\left(A+A^{T}\right)$ where $D_{l}$ and ${\tilde{D}}_{l}$ are two diagonal matrices and $D_{l}\left(i,i\right)=\overset{m}{\underset{j=1}{\sum }}a_{ij}$ and ${\tilde{D}}_{l}\;\left(i,i\right)=\overset{m}{\underset{i=1}{\sum }}a_{ij}$ . Similarly, $L_{d}$ can be computed.

We compute A and B by solving Eq. 24 through an alternating gradient ascent approach.

Finally, lncRNA-disease association score $Y_{fin}\left(i,j\right)$ for each lncRNA-disease pair can be computed by Eq. 25:

$$Y_{fin}=AB^{T}$$ (25)

## 4 Results

### 4.1 Experimental settings

We conduct 5-fold cross validation for 10 times to investigate the performance of LDA-RWLMF. AUC is used to evaluate the prediction accuracy of LDA identification models. AUC is the area under the true positive rate (TPR)-false positive rate (FPR) curve, where TPR and FPR are defined by Eqs 26, 27:

$$TPR=\frac{TP}{TP+FN}$$ (26)

$$FPR=\frac{FP}{TN+FP}$$ (27)

where TP, FP, TN, FN represent the number of true positives, false positives, true negatives, false negatives, respectively. Higher AUC is, better the prediction performance is. In addition, parameters in LDA-RWLMF are set to defaults provided by Peng et al. (2020b). And parameters in the other four comparison LDA prediction methods (LNCSIM1, LNCSIM2, ILNCSIM, and IDSSIM) are set to the same values provided by corresponding methods.

### 4.2 Performance comparison with other methods

To measure the performance of the proposed LDA-RWLMF method, we compare it with four other representative LDA inference approaches on the MNDR dataset. That is, LNCSIM1 (Chen, 2015), LNCSIM2 (Chen, 2015), ILNCSIM (Huang et al., 2016), and IDSSIM (Fan et al., 2020). LNCSIM1 and LNCSIM2 used Laplacian regularized least squares to predict possible LDAs based on disease DAGs and the information content, respectively. ILNCSIM first combined the hierarchical structure of disease DAG and the information content to compute disease similarity and then used Laplacian regularized least squares to infer new LDAs. IDSSIM designed a weighted K nearest neighbor approach to identify potential associations between lncRNAs and diseases by integrating disease semantic similarity and lncRNA functional similarity. Table 1 gives the AUC values of the four LDA identification methods and our proposed LDA-RWLMF on the MNDR dataset.

**TABLE 1:** AUCs of LDA identification approaches on the MNDR dataset.

Dataset	LNCSIM1	LNCSIM2	ILNCSIM	IDSSIM	LDA-RWLMF
the MNDR dataset	0.9251	0.9280	0.9267	0.9302	0.9312

**FIGURE 2:**
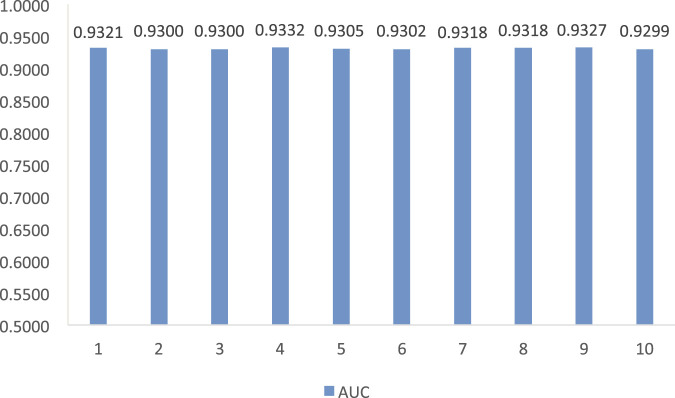
The AUC of LDA-RWLMF from 10 time cross validation (*t* = 1, 2, 3, … , 10).

The results from Table 1 demonstrate that LDA-RWLMF computes the highest AUC compared to LNCSIM1, LNCSIM2, ILNCSIM, and IDSSIM on the MNDR dataset. Figure 2 gives the results of LDA-RWLMF from 10 time cross validation. From Figure 2, we can find that AUC obtain by LDA-RWLMF is relatively steady during 10 time cross validation.

### 4.3 Case study

#### 4.3.1 lncRNA biomarker identification for breast cancer

Breast cancer is the commonest life-threatening cancer in women (Key et al., 2001; Sharma, et al., 2010). lncRNAs play important roles in epigenetic regulation, transcriptional regulation and post-transcriptional regulation and have been potential biomarkers of many diseases. Substantial publications have reported that lncRNAs affect proliferation and apoptosis, invasion and metastasis, and cancer stemness of breast cancer. For example, LSINCT5 and Zfas one can promote the proliferation of breast cancer, HOTAIR suppresses invasion and migration of breast cancer, SOX2OT induces SOX2 expression in breast cancer, and SRA is the expression activator of breast cancer (Sun et al., 2017). We want to conduct case analyses to find possible lncRNA biomarkers for breast cancer based on the proposed LDA-RWLMF model.

In the MNDR dataset, there are 89 lncRNAs that may associate with breast cancer, where 54 lncRNAs have been experimentally validated to associate with the cancer and 35 lncRNAs have unknown associations with it. We use the proposed LDA-RWLMF method to rank the 89 lncRNAs for breast cancer. The results are shown in Tables 2, 3. Table 2 demonstrates the ranking results of the predicted top 48 lncRNAs according to the computed association score with breast cancer on the MNDR dataset. These 48 lncRNAs are known to link to breast cancer on the MNDR dataset and are ranked as top 48.

**TABLE 2:** The rankings of the predicted top 48 lncRNAs according to association with breast cancer on the MNDR dataset.

Rank	lncRNA	Evidence	Rank	lncRNA	Evidence
1	CASC2	Known	25	PVT1	Known
2	DLEU2	Known	26	RMST	Known
3	MIR17HG	Known	27	TRAF3IP2-AS1	Known
4	DSCAM-AS1	Known	28	HCP5	Known
5	SNHG4	Known	29	LINC00271	Known
6	TCL6	Known	30	GHET1	Known
7	XIST	Known	31	SNHG3	Known
8	CBR3-AS1	Known	32	TDRG1	Known
9	MIAT	Known	33	DAOA-AS1	Known
10	CCAT2	Known	34	BACE1-AS	Known
11	SOX2-OT	Known	35	NAMA	Known
12	GAS5	Known	36	BDNF-AS	Known
13	PCA3	Known	37	SNHG11	Known
14	MALAT1	Known	38	UCA1	Known
15	BANCR	Known	39	SNHG16	Known
16	WT1-AS	Known	40	MIR100HG	Known
17	PANDAR	Known	41	H19	Known
18	HNF1A-AS1	Known	42	TERC	Known
19	HAR1B	Known	43	MEG3	Known
20	CCDC26	Known	44	SPRY4-IT1	Known
21	BCAR4	Known	45	DANCR	Known
22	PDZRN3-AS1	Known	46	KCNQ1OT1	Known
23	HIF1A-AS2	Known	47	IFNG-AS1	Known
24	CRNDE	Known	48	HOTAIR	Known

**TABLE 3:** The rankings of the remaining 41 lncRNAs according to association with breast cancer on the MNDR dataset.

Rank	lncRNA	Evidence	Rank	lncRNA	Evidence
49	HULC	PMID: 31824174, 33107484, 33745450	70	ZFAT-AS1	Unconfirmed
50	CCAT1	Known	71	PTENP1	PMID: 28731027, 29085464, 29212574, 31196157
51	NPTN-IT1	Unconfirmed	72	HIF1A-AS1	Unconfirmed
52	PCAT1	PMID: 32853955, 28989584, 33850635, 32220602	73	SRA1	Known
53	HAR1A	PMID: 26942882	74	MINA	Unconfirmed
54	LSINCT5	Known	75	DLEU1	Known
55	TUG1	PMID: 28950664, 27848085, 30098551, 33380806	76	PSORS1C3	Unconfirmed
56	MIR155HG	Unconfirmed	77	LINC00032	Unconfirmed
57	DGCR5	PMID: 32521856	78	WRAP53	Unconfirmed
58	IGF2-AS	PMID: 33175607	79	7SK	Unconfirmed
59	BCYRN1	Known	80	RRP1B	Unconfirmed
60	EPB41L4A-AS1	PMID: 35181612	81	MYCNOS	Unconfirmed
61	PINK1-AS	Unconfirmed	82	PRINS	Unconfirmed
62	DNM3OS	Unconfirmed	83	ATP6V1G2-DDX39B	Unconfirmed
63	ADAMTS9-AS2	PMID: 30840279	84	MKRN3-AS1	Unconfirmed
64	MIR31HG	lncRNADisease	85	NRON	Unconfirmed
65	BOK-AS1	Unconfirmed	86	MESTIT1	Unconfirmed
66	ESRG	Unconfirmed	87	LINC00162	Unconfirmed
67	KCNQ1DN	Unconfirmed	88	DISC2	Unconfirmed
68	ATXN8OS	PMID: 31173245, 33385064, 33477683	89	SCAANT1	Unconfirmed
69	CDKN2B-AS1	Known			

Table 3 gives the rankings of the remaining 41 lncRNAs according to the association scores with breast cancer on the MNDR dataset. Among all lncRNAs unknown to associate with breast cancer on the MNDR dataset, lncRNA HULC is predicted to link to breast cancer with the highest association scores. Shi et al. (2016) observed that HULC can act as an oncogene biomarker in triple-negative breast cancer and as an independent possible poor prognostic factor in patients suffered from triple-negative breast cancer. Wang et al. (2019) found that HULC can promote the development of breast cancer through regulating the expression of LYPD1. Gavgani et al. (2020) investigated that the HULC knockdown can induce apoptosis and suppress cellular migration in breast cancer cells.

PCAT1 may link to breast cancer with the ranking of three among all lncRNAs unknown to associate with breast cancer on the MNDR dataset. Several studies have reported that PCAT1 can associate with breast cancer although its association with the cancer on the MNDR dataset is unobserved. Abdollahzadeh et al. (2020) reported that the altered regulation of PCAT1 may play crucial roles in the development and pathogenesis of breast cancer. Sarrafzadeh et al. (2017) assessed the expression of PCAT-1 through real-time reverse transcription polymerase chain reaction in breast tumor samples from 47 breast cancer patients and found that PCAT-1 may involve in the pathogenesis of breast cancers. Wang et al. (2021a) observed that PCAT-1 can facilitate breast cancer progression by binding to RACK1 and thus boosting oxygen-independent stability of HIF-1α. Tang et al. (2022) detect that PCAT1 can regulate the expression of PITX2 in breast cancer.

**FIGURE 3:**
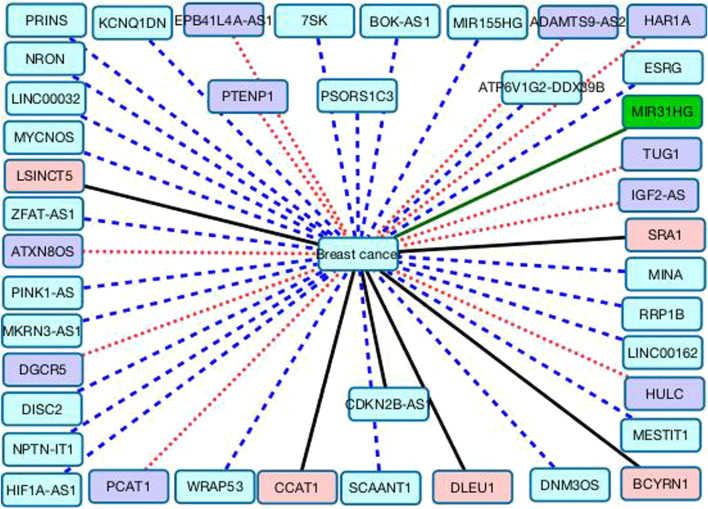
The associations between the remaining 41 lncRNAs and breast cancer.

In addition, we predict that nephronectin intronic transcript 1 (NPTN-IT1, also known as lncRNA-LET) may have relationship with breast cancer. NPTN-IT1 has been reported to associate with bladder cancer through attenuating the expression of the target of miR-145 and ILF3 in bladder cancer (Zhang et al., 2021b). It was significantly down-regulated in multiple tumor tissues of colorectal cancer. It also has a regulation role in hypoxia signaling of hepatocellular carcinoma (Sun et al., 2013) and was highly expressed in HepG2 cells (Kong et al., 2019b). We hope that association between three lncRNAs (HULC, NPTN-IT1, and PCAT1) and breast cancer can be validated through wet experiments. Figure 3 shows the associations between the 41 lncRNAs that are ranked as the last 41 and breast cancer. Black solid lines represent known LDAs in the MNDR database. Green solid lines represent LDAs that can be observed in the lncRNA disease database. Red dots lines represent LDAs that are predicted to be potential lncRNA biomarkers of breast cancer and can be confirmed by related publications. Blue equal dash lines represent unknown LDAs.

#### 4.3.2 lncRNA biomarker identification for colorectal cancer

Colorectal cancer is a heterogeneous disease. It has high morbidity and mortality. lncRNAs demonstrate dense associations with colorectal cancer. In this study, we conduct case analyses to identify possible lncRNA biomarkers for colorectal cancer based on LDA-RWLMF. In the MNDR dataset, 89 lncRNAs possibly associate with colorectal cancer, where 55 lncRNAs have been validated to be the biomarkers of the cancer and remaining 34 lncRNAs have not been validated. We use LDA-RWLMF to compute the association scores between all 89 lncRNAs and colorectal cancer and rank the 89 lncRNAs for colorectal cancer. The results are shown in Tables 4, 5. Table 4 shows the rankings of the identified top 50 lncRNAs according to the computed association score with colorectal cancer on the MNDR dataset. The 50 lncRNAs are known to associate with colorectal cancer on the MNDR dataset and are ranked as top 50.

**TABLE 4:** The rankings of the identified top 50 lncRNAs associated with colorectal cancer on the MNDR dataset.

Rank	lncRNA	Evidence	Rank	lncRNA	Evidence
1	SOX2-OT	Known	26	NAMA	Known
2	DLEU2	Known	27	WT1-AS	Known
3	CASC2	Known	28	TDRG1	Known
4	TCL6	Known	29	GHET1	Known
5	TRAF3IP2-AS1	Known	30	CRNDE	Known
6	DSCAM-AS1	Known	31	XIST	Known
7	GAS5	Known	32	MALAT1	Known
8	MIR17HG	Known	33	RMST	Known
9	HAR1B	Known	34	SNHG3	Known
10	CCDC26	Known	35	BACE1-AS	Known
11	CBR3-AS1	Known	36	MIR100HG	Known
12	PANDAR	Known	37	IFNG-AS1	Known
13	MIAT	Known	38	DANCR	Known
14	SNHG4	Known	39	SNHG16	Known
15	HIF1A-AS2	Known	40	SNHG11	Known
16	HNF1A-AS1	Known	41	TERC	Known
17	PCA3	Known	42	KCNQ1OT1	Known
18	BANCR	Known	43	MEG3	Known
19	LINC00271	Known	44	HULC	Known
20	PDZRN3-AS1	Known	45	UCA1	Known
21	CCAT2	Known	46	SPRY4-IT1	Known
22	BCAR4	Known	47	PCAT1	Known
23	DAOA-AS1	Known	48	HOTAIR	Known
24	BDNF-AS	Known	49	PVT1	Known
25	HCP5	Known	50	CCAT1	Known

**TABLE 5:** The rankings of the remaining 41 lncRNAs according to association with breast cancer on the MNDR dataset.

Rank	lncRNA	Evidence	Rank	lncRNA	Evidence
51	HAR1A	Unconfirmed	71	ZFAT-AS1	Unconfirmed
52	NPTN-IT1	known	72	SRA1	Unconfirmed
53	TUG1	known	73	PSORS1C3	Unconfirmed
54	IGF2-AS	PMID: 32853944, 30581274	74	HIF1A-AS1	Unconfirmed
55	LSINCT5	known	75	MINA	Unconfirmed
56	DGCR5	PMID: 31452812	76	LINC00032	Unconfirmed
57	H19	known	77	WRAP53	Unconfirmed
58	EPB41L4A-AS1	PMID: 32557646	78	DLEU1	Unconfirmed
59	MIR155HG	PMID: 34562123,31228357	79	RRP1B	Unconfirmed
60	CDKN2B-AS1	known	80	7SK	Unconfirmed
61	MIR31HG	PMID: 30447009,35733512,34485123	81	PRINS	Unconfirmed
62	ESRG	PMID: 34896077	82	MYCNOS	Unconfirmed
63	BCYRN1	PMID: 30114690,32944001,31773686	83	ATP6V1G2-DDX39B	Unconfirmed
64	BOK-AS1	Unconfirmed	84	MKRN3-AS1	Unconfirmed
65	PINK1-AS	Unconfirmed	85	NRON	Unconfirmed
66	KCNQ1DN	Unconfirmed	86	SCAANT1	Unconfirmed
67	ATXN8OS	Unconfirmed	87	DISC2	Unconfirmed
68	DNM3OS	Unconfirmed	88	MESTIT1	Unconfirmed
69	PTENP1	Unconfirmed	89	LINC00162	Unconfirmed
70	ADAMTS9-AS2	Unconfirmed			

Table 5 gives the rankings of the remaining 39 lncRNAs according to the association scores with colorectal cancer on the MNDR dataset. Among all lncRNAs unknown association with colorectal cancer on the MNDR dataset, lncRNA HAR1A is inferred to link to colorectal cancer with the highest association scores. HAR1A is a favorable prognostic biomarker for patients. Shi et al. (2019) analyzed the expression profiles of HAR1A using RT-qPCR and found its expression level was significantly lower in hepatocullular cancer. Chen et al. (2020) have still reported that the HAR1A expression levels were reduced in hepatocellular carcinoma tissues.

**FIGURE 4:**
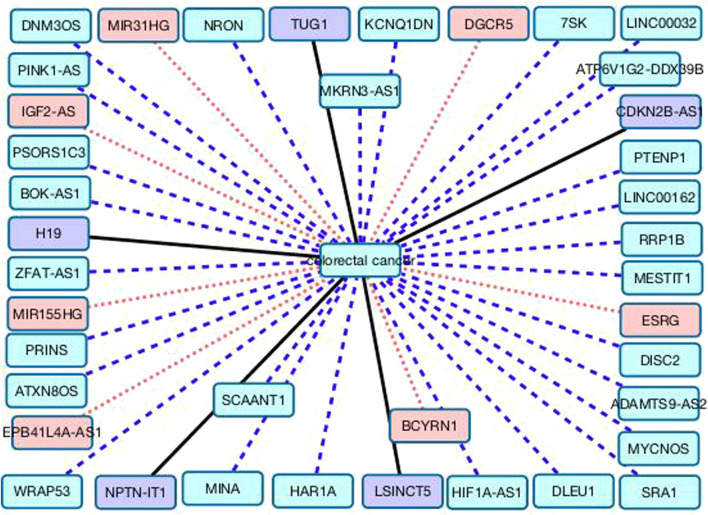
The associations between the remaining 39 lncRNAs and colorectal cancer.

Figure 4 gives the associations between the remaining 39 lncRNAs and colorectal cancer. Black solid lines represent known LDAs in the MNDR database. Red dots lines represent LDAs that are predicted to be potential lncRNA biomarkers of breast cancer and can be confirmed by related publications. Blue equal dash lines represent unknown LDAs.

## 5 Discussion and conclusion

Breast cancer and colorectal cancer are the most frequent cancers with high mortality rates. They demonstrate very high heterogeneity at molecular and clinical levels. With the fast development of next generation sequencing technologies, we can more accurately characterize the human genome. lncRNAs act mainly as gene expression regulators. The dysregulation of lncRNAs may destroy the normal transcriptional landscape and thus cause malignant transformation. In addition, their highly specific expression and functional tertiary structure force them to be as promising diagnostic biomarkers and potential targets for various diseases including breast cancer and colorectal cancer.

In this study, we proposed a computational lncRNA-disease association method (LDA-RWLMF) to identify potential biomarkers for breast cancer and colorectal cancer. First, a random walk with restart method was designed to extract negative LDAs. Second, a logistic matrix factorization model was explored to infer possible associations between lncRNAs and diseases. Finally, all lncRNAs are ranked according to association scores with breast cancer and colorectal cancer on the MNDR dataset.

We conduct 5-fold cross validation for 10 times to compare LDA-RWLMF with state-of-the-art LDA prediction models on the MNDR dataset, that is, LNCSIM1, LNCSIM2, ILNCSIM, and IDSSIM. The results show that LDA-RWLMF computes the best AUC values of 0.9312. We predict that lncRNAs (HULC, NPTN-IT1, and PCAT1) may be possible biomarkers of breast cancer and colorectal cancer.

Our proposed LDA-RWLMF method has two disadvantages. First, it extracted credible negative LDA samples. In the area of association prediction, there are no negative association samples because of the limitation of biomedical experiments, which causes relatively poor performance. Thus, we designed a negative LDA extraction method based on PU learning. Second, the logistic matrix factorization model can effectively discover possible associations between two biological entities. Thus, we used the model to identify new LDAs. In addition, diseases and lncRNAs exhibit abundant biological features. In this study, we failed to consider these diverse features. In the future, we will further integrate more biological information to improve LDA prediction.

In the future, we will further design more effective negative sample screening method based on positive-unlabeled learning. In addition, we will also develop deep learning model for LDA prediction. We anticipate that the proposed LDA-RWLMF method can help design therapeutic regimens for personalized treatment of breast cancer and colorectal cancer and thus opportunely inhibit its recurrence.

## Data Availability

The original contributions presented in the study are included in the article/supplementary material, further inquiries can be directed to the corresponding author.
